# Haplotype defined by the *MLH1*-93G/A polymorphism is associated with *MLH1* promoter hypermethylation in sporadic colorectal cancers

**DOI:** 10.1186/1756-0500-7-835

**Published:** 2014-11-24

**Authors:** Yasuyuki Miyakura, Makiko Tahara, Alan T Lefor, Yoshikazu Yasuda, Kokichi Sugano

**Affiliations:** Department of Surgery, Jichi Medical University, 3311-1, Shimotsuke, Tochigi 329-0498 Japan; Oncogene Research Unit/Cancer Prevention Unit, Tochigi Cancer Center Research Institute, 4-9-13, Yohnan, Utsunomiya, Tochigi 320-0834 Japan

**Keywords:** MSI, SNP, *MLH1*, Methylation, Colorectal cancer, Haplotype

## Abstract

**Background:**

Methylation of the *MLH1* promoter region has been suggested to be a major mechanism of gene inactivation in sporadic microsatellite instability-positive (MSI-H) colorectal cancers (CRCs). Recently, single-nucleotide polymorphism (SNP) in the *MLH1* promoter region (*MLH1*-93G/A; rs1800734) has been proposed to be associated with *MLH1* promoter methylation, loss of MLH1 protein expression and MSI-H tumors. We examined the association of *MLH1*-93G/A and six other SNPs surrounding *MLH1*-93G/A with the methylation status in 210 consecutive sporadic CRCs in Japanese patients.

**Methods:**

Methylation of the *MLH1* promoter region was evaluated by Na-bisulfite polymerase chain reaction (PCR)/single-strand conformation polymorphism (SSCP) analysis. The genotype frequencies of SNPs located in the 54-kb region surrounding the *MLH1*-93G/A SNP were examined by SSCP analysis.

**Results:**

Methylation of the *MLH1* promoter region was observed in 28.6% (60/210) of sporadic CRCs. The proportions of *MLH1*-93G/A genotypes A/A, A/G and G/G were 26% (n = 54), 51% (n = 108) and 23% (n = 48), respectively, and they were significantly associated with the methylation status (p = 0.01). There were no significant associations between genotype frequency of the six other SNPs and methylation status. The A-allele of *MLH1*-93G/A was more common in cases with methylation than the G-allele (p = 0.0094), especially in females (p = 0.0067). In logistic regression, the A/A genotype of the *MLH1*-93G/A SNP was shown to be the most significant risk factor for methylation of the *MLH1* promoter region (odds ratio 2.82, p = 0.003). Furthermore, a haplotype of the A-allele of rs2276807 located -47 kb upstream from the *MLH1*-93G/A SNP and the A-allele of *MLH1*-93G/A SNP was significantly associated with *MLH1* promoter methylation.

**Conclusions:**

These results indicate that individuals, and particularly females, carrying the A-allele at the *MLH1*-93G/A SNP, especially in association with the A-allele of rs2276807, may harbor an increased risk of methylation of the *MLH1* promoter region.

**Electronic supplementary material:**

The online version of this article (doi:10.1186/1756-0500-7-835) contains supplementary material, which is available to authorized users.

## Background

Microsatellite instability (MSI) is a form of genomic instability that can be detected as changes in the length of repetitive microsatellite sequences. MSI occurs in the majority of tumors from patients with Lynch syndrome carrying germline mutations in the DNA mismatch repair (*MMR*) genes [1]. MSI also occurs in approximately 15-20% of sporadic colorectal cancers (CRCs) [1, 2]. Methylation of the *MLH1* promoter region has been suggested to be a major mechanism of gene inactivation in sporadic MSI-positive CRCs [3]. Bi-allelic methylation of the *MLH1* promoter region induced transcriptional silencing in cell lines showing MSI and this epigenetic mechanism of gene inactivation is analogous to Knudson’s two-hit hypothesis [4, 5].

We previously reported that all CpG sites in a 1-kb region encompassing the *MLH1* promoter were methylated (designated as full methylation) in CRCs showing high-frequency MSI (MSI-H) [6]. Full methylation was particularly observed in proximal colon cancers in females older than 70 years old and led to the transcriptional silencing of the *MLH1* gene and the MSI-H phenotype [7]. Methylation of the *MLH1* promoter region was also observed in 20% of MSI-negative CRCs, although all of these cases displayed partial methylation (limited to the most upstream region of the *MLH1* promoter); MLH1 protein expression was maintained in such cases showing partial methylation. Thus, there is an important correlation between the methylation status of the *MLH1* promoter and the development of sporadic MSI-positive CRCs.

Single-nucleotide polymorphisms (SNPs) are the most common type of human genetic variation and occur at about every 1000 bases in the human genome. Numerous studies are now ongoing to assess their possible associations with human diseases or phenotypes [8–10]. SNPs in the promoter region have been shown to influence gene transcription [11]. G/A SNP -93 bp upstream of the translation start site was identified in the *MLH1* gene promoter region [*MLH1*-93G/A SNP (rs1800734)] [12]. The *MLH1*-93G/A SNP has recently been proposed to have an association with CRCs, ovarian and endometrial cancer [13–16]. In particular, it was shown to be associated with *MLH1* promoter methylation, loss of MLH1 protein expression and MSI-H tumors [14, 16–21]. However, these results are based largely on analyses of cancers in Caucasian populations. Allelic frequencies of the *MLH1*-93G/A SNP differ between Caucasians and Japanese, and the frequency of the A-allele in Caucasians (20-23%) is lower than that in Japanese (46%) [12, 16, 20].

The purpose of this study was to address the association between the *MLH1*-93G/A SNP and methylation status in the *MLH1* promoter region in a series of sporadic CRCs in a Japanese population. Furthermore, we also examined the association between six other SNPs located in the 54-kb region surrounding the *MLH1*-93G/A SNP and methylation status in the *MLH1* promoter region. We report here that the *MLH1*-93G/A SNP was significantly associated with *MLH1* promoter methylation in a Japanese population. Furthermore, a haplotype comprising the A-allele of rs2276807 and the A-allele of *MLH1*-93G/A SNP showed a significant association with *MLH1* promoter methylation. This haplotype may be associated with an increased risk for methylation-positive CRCs, especially in females.

## Methods

### Tissue samples

Tumor tissues and corresponding normal mucosa were obtained from 210 consecutive CRC patients who underwent surgery from 1996 through 1998 at Jichi Medical University Hospital. Genetic analyses were carried out in the Oncogene Research Unit/Cancer Prevention Unit, Tochigi Cancer Center Research Institute. As all samples were derived from archived tissue samples, they were coded anonymously, prior to the analysis of methylation status and genotyping of the vicinity of the *MLH1* promoter regions, according to the Ethical Guidelines for Human Genome/Gene Analysis Research (Ministry of Education, Culture, Sports, Science and Technology, Ministry of Health, Labour and Welfare and Ministry of Economy, Trade and Industry, March 29, 2001) and the study protocol was approved by the institutional review board of each institution. Normal mucosa was obtained from the surgical margin of the resected specimen before sampling the tumor tissue to avoid contamination by tumor cells. Kindred in whom the Lynch syndrome was suspected were excluded from this study, since patients with at least one CRC among first-degree relatives were not included. Tumors were staged according to a modified version of Dukes classification [22]. DNA was extracted from fresh tumor material by a standard procedure using proteinase K digestion and subsequent phenol-chloroform extraction. Methylation status and the genotype of the *MLH1* promoter region were also examined in peripheral blood lymphocytes (PBLs) from 100 anonymous samples obtained from normal healthy donors over 50 years of age undergoing routine health checkups.

### Analysis of MSI

Genomic DNA was subjected to PCR amplification at two mononucleotide microsatellite repeat loci, BAT26 and BAT25. The BAT26 locus contains a 26-repeat adenine tract and is located in intron 5 of the *MSH2* gene, whereas the BAT25 locus contains a 25-repeat thymine tract located in intron 16 of the *c*-*kit* oncogene. Both loci have been shown to be sensitive markers of MSI, which manifests as alteration in the size of the respective mononucleotide repeats in tumor DNA. PCR reactions were performed as described previously [6]. When either BAT26 or BAT25 showed MSI, we examined 7 microsatellite repeat loci, D2S123, D5S346, D17S250, MSH3, MSH6, TGFBR2 and BAX, according to the recommendations of the Bethesda guidelines [23]. Tumors were classified as MSI-H when ≥30% of the 9 markers showed MSI. Low-frequency MSI (MSI-L) is classified as MSI-negative.

### Methylation analysis of the *MLH1*promoter region

**Figure 1:**
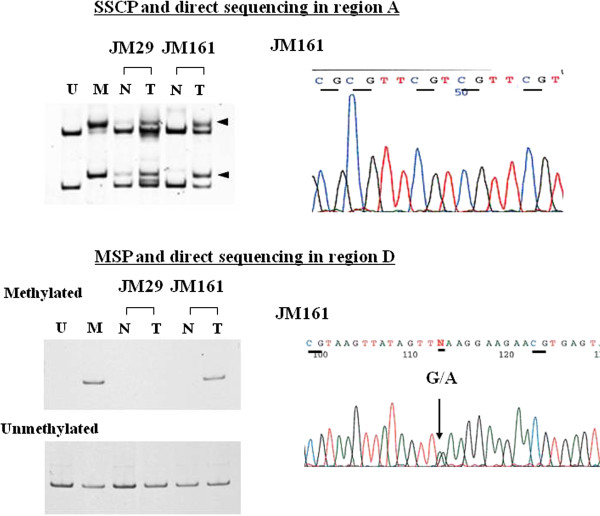
**BiPS and MSP analysis of** ***MLH1*** **promoter region.** Upper panel: BiPS analysis of *MLH1* promoter region A is shown. U indicates control unmethylated normal DNA; M indicates control methylated DNA. Cancer tissue (T) from cases JM29 and JM161 displayed a methylated band in region A. After SSCP analysis, the extra bands were cut from the electrophoresed gels, reamplified by PCR and sequenced by the dideoxy sequencing procedure. Results of the direct sequencing are shown in the right panel. Sequences (JM161) with methylated cytosines of CpG dinucleotides in region A are shown. Lower panel: MSP analysis of *MLH1* promoter region D and direct sequencing of the amplified DNA fragments. U, control unmethylated normal DNA; M, control methylated DNA. Tumor tissue (T) from case JM161 (full methylation case) also showed a methylated band in promoter region D. In contrast, tumor tissue (T) from case JM29 (partial methylation case) did not show a methylated band in the same region.

Na-bisulfite PCR/single-strand conformation polymorphism (SSCP) (BiPS) analysis was performed as described previously [6, 24]. With the adenine residue at the initiation codon numbered as +1 nt, the *MLH1* promoter (-755 to +86) was divided into five regions [region A (from -755 to -574, containing 23 CpG sites), B (from -597 to -393, 12 CpG sites), C (from -420 to -188, 16 CpG sites), D (from -286 to -53, 13 CpG sites) and E (from -73 to +86, 13 CpG sites)] and amplified with 5 sets of PCR primers. Sequences of PCR primers were as reported previously [6]. Each primer set was designed to anneal to both methylated and unmethylated DNA sequences and the amplicons could be separated by SSCP analysis depending on their methylation status (Figure 1). Amplified DNA fragments were visualized using SYBR^®^ Gold nucleic acid gel stain (Cosmo Bio Co., Tokyo, Japan) and scanned with a Fluorescent Image Analyzer Model FLA-3000G (Fuji Photo Film Co., Tokyo, Japan). When the bands showed mobility shifts, they were cut from the gel, reamplified and directly sequenced using an ABI 310 PRISM™ sequencer (Perkin-Elmer Co., Branchburg, NJ) with a Big-Dye Terminator Cycle Sequencing Ready Reaction Kit™ (Perkin-Elmer Co., Branchburg, NJ). Methylation profiles were classified as follows: full methylation if all CpG sites in regions A to E showed methylation, partial methylation if some CpG sites, in any region, showed methylation, and no methylation if all CpG sites in any region did not show methylation. Methylation status of the *MLH1* promoter region D was also analyzed by methylation-specific PCR (MSP) and the allelic status of methylation was examined by direct sequencing of the *MLH1*-93G/A SNP in region D (Figure 1) [7].

### Analysis of the genotype frequencies of the *MLH1*promoter region

The genotype frequencies of the *MLH1*-93G/A SNP were examined by SSCP analysis using an ALFexpress DNA sequencer (Pharmacia, Tokyo, Japan). Briefly, primer sequences were 5′Cy5-gacgaagagacccagcaaccc3′ (forward) and 5′tagatgctcaacggaagtgc 3′ (reverse). The 5′-terminus of the forward primer was labeled with indodicarbocyanine (Cy5) fluorescent dye. PCR reactions were performed as described previously [7]. The data were analyzed using the software package Fragment Manager™ (Pharmacia, Tokyo, Japan).

### Analysis of six SNPs surrounding the *MLH1*-93G/A SNP

**Figure 2:**
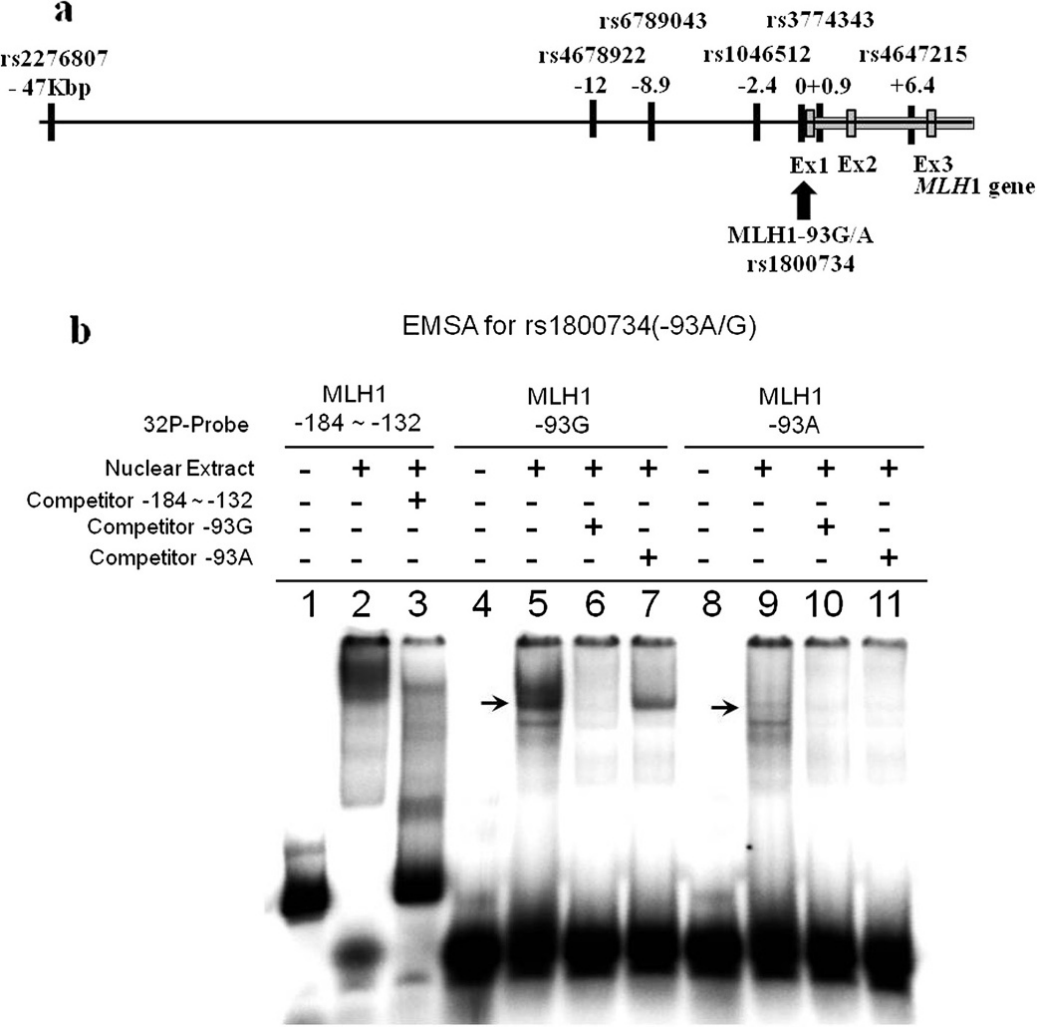
**Map of six SNPs and EMSA for rs1800734. a**. Map of six SNPs, rs2276807 (C/A), rs4678922 (A/T), rs6789043 (T/C), rs1046512 (C/A), rs3774343 (G/A) and rs4647215 (C/A), located in the 54-kb region surrounding the *MLH1*-93G/A SNP. **b**. EMSA using nuclear extracts from HeLa cells. The competition assay was performed with ^32^P-labeled MLH1-184 to -132 and unlabeled MLH1-184 to -132 oligomer (lanes 1–3). A shifted band was observed in the presence of HeLa nuclear extract, which was blocked in the presence of excess unlabeled competitor (lanes 2 and 3). The competition assay was performed with matched sets of DNA oligomers homologous to each genotype of the *MLH1*-93G/A SNP (lanes 5–7, 9–11). When a ^32^P-labeled MLH-93G probe was mixed with nuclear extract from HeLa cells, a shifted band was blocked by the competitor oligomer of MLH1-93G, but not MLH1-A (lanes 5–7). When ^32^P-labeled MLH-93A probe was mixed with the nuclear extract from HeLa cells, a shifted band was not detected (lanes 9–11).

The genotype frequencies of the following six SNPs, rs2276807 (C/A), rs4678922 (A/T), rs6789043 (T/C), rs1046512 (C/A), rs3774343 (G/A) and rs4647215 (C/A) (Figure 2a), located in the 54-kb region surrounding the *MLH1*-93G/A SNP, were examined by SSCP analysis. The locations of the SNPs and the PCR primer sequences are described in Additional file 1: Table S1. Each PCR was carried out using 0.1 μg of genomic DNA as a template, 10×PCR buffer, 1.25 mM dNTP, 10 μM each primer and 0.625 units of *Taq* DNA polymerase in a total volume of 25 μL. The PCR conditions were as follows: heat denaturation at 95°C for 12 min, 30 cycles of PCR comprising 95°C for 30 sec, an annealing step with different temperatures, namely, 55°C (rs4678922, rs6789043, rs1046512, rs3774343), 63°C (rs2276807) or 68°C (rs4647215), for 30 sec, and an extension step at 72°C for 30 sec, followed by a final extension at 72°C for 10 min. Conditions of the SSCP analysis are described in Additional file 1: Table S1. The data were analyzed using the software package Fragment Manager™ (Pharmacia, Tokyo, Japan).

### Electrophoretic mobility shift assay (EMSA)

EMSA was performed using the Gelshift™ Kit (Active Motif, Carlsbad, CA) (Figure 2b). The sequences of ^32^P-labeled complementary oligonucleotide pairs and complementary competitor oligonucleotide pairs corresponding to the *MLH1* promoter sequence were as follows: MLH-184 to -132: 5′-ACCCACAGAGTTGAGAAATTTGACTGGCATTCAAGCTGTCCAATCAATAGCTG-3′ [12], *MLH1*-93G: 5′-AGCTACAGCTGAAGGAAGAA-3′, and *MLH1*-93A: 5′-AGCTACAGCTAAAGGAAGAA-3′. After the reaction mixtures with and without an excess of unlabeled competitor and HeLa cell nuclear extract (HeLa Nuclear Extract™; Activemotif, Carlsbad, CA) were incubated for 20 min on ice, ^32^P-labeled oligonucleotides were added as a probe. The binding reactions were carried out according to the manufacturer’s instruction manual. After the binding reaction, the DNA protein complexes were resolved by electrophoresis in a 5% non-denaturing acrylamide gel chilled at 4°C using a circulating water bath. After electrophoresis, the gel was dried and scanned with a Fluorescent Image Analyzer Model FLA-3000G (Fuji Photo Film Co., Tokyo, Japan).

### Statistical analysis

Statistical analysis was carried out using Pearson’s chi-square (Χ^2^) test or Student’s t-test. Multivariate logistic regression analysis was performed using Stat View 5.0 statistical package (SAS Institute Inc., Cary, NC) to identify independent variables associated with the presence of *MLH1* promoter methylation. The included variables were *MLH1*-93G/A genotype (A/A vs. A/G + G/G), tumor location (right vs. left), gender (female vs. male) and age at onset (more than 70 vs. less than 70). Absence of departure from the Hardy-Weinberg equilibrium was calculated by the asymptotic Χ^2^ goodness of fit test. The haplotypes of the seven SNPs were analyzed using SNPAlyze software ver. 5.0 (Dynacom, Chiba, Japan) and a case–control study was performed to examine the relationship between methylation-positive and methylation-negative subjects. Permutation analysis was used to determine the empirical significance and to calculate the probability (p) values based on 10,000 replications. The global p-values represent the overall significance using the Χ^2^-test when the observed versus expected frequencies of all haplotypes are considered together.

The individual haplotypes were tested for association by grouping all other haplotypes together and applying the Χ^2^-test with one degree of freedom. P-values <0.05 were considered statistically significant.

## Results

### Comparison of clinicopathological and molecular features with methylation status of the *MLH1*promoter region

The clinicopathological background, MSI status and the association of these variables with methylation status are summarized in Table 1. The category of methylation-positive includes both full methylation (n = 13) and partial methylation (n = 47). There were significant differences between methylation-positive (n = 60) and -negative groups (n = 150) with respect to gender, tumor location, histologic type and tumor size (Table 1). Methylation-positive tumors showed a marked preponderance in females (p = 0.007) and the proximal colon (p = 0.004). The proportion of well-differentiated adenocarcinomas was lower in the methylation-positive group (p = 0.04). The average tumor size was significantly larger in methylation-positive tumors (60.0 ± 28.9 mm) than in methylation-negative ones (52.2 ± 22.4 mm) (p = 0.04). Regarding MSI status, the frequency of MSI-H was significantly higher in the methylation-positive group (p < 0.0001).

**Table 1:** **Association between clinicopathological features and MSI status and methylation status of the** ***MLH1*** **promoter region in sporadic colorectal cancers**

	Methylation-positive				Methylation-negative	p values ^4^
	Full methylation	Partial methylation	p values ^3^	Total (Full + Partial methylation)	(No methylation)	
Numbers of cases	13	47		60	150	
Age at onset	72.8 ± 10.6	64.5 ± 10.1	0.012	66.3 ± 10.7	63.9 ± 11.1	NS
Gender						
Male	3	25	NS(0.054)	28	100	0.007
Female	10	22		32	50	
Tumor location^1^						
Right	13	15	<0.0001	28	39	0.004
Left	0	32		32	110	
Histological type^2^						
well	6	24	NS	30	105	0.04
mod	3	19		22	36	
muc	2	3		5	5	
por	2	1		3	4	
Dukes						
A/B	1/10	2/27	NS	3/37	8/75	NS
C/D	1/1	14/4		15/5	51/16	
Tumor size (mm)	66.4 ± 35.5	58.2 ± 26.9	NS	60.0 ± 28.9	52.2 ± 22.4	0.04
MSI-H	13	2	<0.0001	15	4	<0.0001

^1^right: cecum, ascending colon and transverse colon, left: descending colon, sigmoid colon and rectum.

^2^well: well-differentiated adenocarcinoma, mod: moderately differentiated adenocarcinoma, por: poorly differentiated adenocarcinoma, muc: mucinous adenocarcinoma.

^3^P values were analyzed using Pearson’s chi-square test or Student’s t-test between cases with full methylation and cases with partial methylation. NS: not significant. P values of <0.05 were statistically significant.

^4^P values were analyzed using Pearson’s chi-square test or Student’s t-test between methylation-positive cases (full methylation + partial methylation) and methylation-negative cases. NS: not significant. P values of <0.05 were statistically significant.

In the methylation-positive group, there were significant differences between patients showing full methylation (n = 13) and partial methylation (n = 47) with regard to age at onset, tumor location and MSI status. The average age at onset was significantly higher in patients with full methylation than in those with partial methylation (72.8 ± 10.6 y/o vs. 64.5 ± 10.1 y/o, p = 0.012). All tumors with full methylation developed in the proximal colon and showed MSI-H (13/13, 100%), while those with partial methylation were more prevalent in the distal colon (32/47, 68.1%) and MSI-H was observed less frequently than in those with full methylation (2/47, 4.3% vs. 13/13, 100%, p < 0.0001).

### Comparison of *MLH1*genotypes with methylation status of the *MLH1*promoter region

Genotypes and allelic frequencies of the *MLH1*-93G/A SNP were examined in 210 sporadic CRC patients and 100 PBL samples from normal donors, and the results were compared with previous studies carried out in cohorts of different populations. The allelic frequency for each allele was nearly 50% in the normal donors, other Japanese and Korean cohorts, while the frequency of the G-allele (78–80%) was higher than that of the A-allele (20–22%) in Caucasians [12, 16, 20, 25]. In each study, observed genotype frequencies were consistent with the expected frequencies according to the Hardy-Weinberg equilibrium.

In the 210 sporadic CRCs, the proportions of genotypes A/A, A/G and G/G were 26% (n = 54), 51% (n = 108) and 23% (n = 48), respectively (Table 2). There were no significant associations between genotypes and clinicopathological parameters such as age at onset, sex, tumor location, Dukes classification and histologic type.

**Table 2:** **Association between methylation status of the** ***MLH1*** **promoter region and genotypes of the** ***MLH1***-**93G**/**A SNP in sporadic colorectal cancers**

<Total cases>	Genotype				Allelic frequency			
	A/A (n = 54)	A/G (n = 108)	G/G (n = 48)	p values	A	G	RR	p values
Methylation-positive	24	26	10	0.0103^1^	74	46	1.303	0.0094^1^
Full methylation	4	8	1		24	10		
Partial methylation	20	18	9	0.0229^2^	58	36		0.0044^2^
Methylation-negative	30	82	38		142	158		
(No methylation)								
<Female cases>	Genotype	Allelic frequency						
	A/A (n = 23)	A/G (n = 37)	G/G (n = 22)	p values	A	G	RR	p values
Methylation-positive	14	13	5	0.0259^1^	41	23	1.525	0.0067^1^
Full methylation	4	5	1		13	7		
Partial methylation	10	8	4	NS^2^	28	16		0.0223^2^
Methylation-negative	9	24	17		42	58		
(No methylation)								
<Male cases>	Genotype	Allelic frequency						
	A/A (n = 31)	A/G (n = 71)	G/G (n = 26)	p values	A	G	RR	p values
Methylation-positive	10	13	5	NS^1^	33	23	1.179	NS^1^
Full methylation	0	3	0		3	3		
Partial methylation	10	10	5	NS^2)^	30	20		NS^2)^
Methylation-negative	21	58	21		100	100		
(No methylation)								

^1^P values were analyzed using Pearson’s chi-square test between methylation-positive (full + partial) and methylation-negative cases (no methylation). NS: not significant. P values of <0.05 were statistically significant.

^2^P values were analyzed using Pearson’s chi-square test between full, partial and methylation-negative cases (no methylation). NS: not significant. P values of <0.05 were statistically significant.

The proportions of the *MLH1* genotypes were compared with methylation-positive and methylation-negative cases or cases of full methylation, partial methylation and no methylation of the *MLH1* promoter region (Table 2), and were significantly associated with methylation status (methylation-positive vs. methylation-negative, p = 0.0103; full methylation vs. partial methylation vs. no methylation, p = 0.0229). Allelic frequencies of the *MLH1*-93G/A SNP were also compared with methylation status of the *MLH1* promoter region. The A-allele was more common in methylation-positive cases (RR 1.303, p = 0.0094) and was also more common in subjects with full methylation and partial methylation than the G-allele (p = 0.0044). In an analysis of cases subdivided by gender, the proportions of the *MLH1* genotypes were significantly associated with methylation status in females (methylation-positive vs. methylation-negative, p = 0.0259), but not in males. Furthermore, allelic frequencies of the A-allele were also significantly associated with methylation status in females (RR 1.525, p = 0.0067).

The clinical characteristics, pathological features and MSI status based on methylation status (full, partial or none) were compared according to three genotypes of the *MLH1*-93G/A SNP (Additional file 1: Table S2). Patients who had tumors with full methylation were older and included a marked preponderance of females (compared to no methylation), right-sided lesions and MSI-H compared to those with partial and no methylation in genotype A/A. In contrast, patients who had tumors with full methylation were significantly different regarding tumor location, pathological type (compared to no methylation) and MSI status compared to those with partial and no methylation in genotype A/G. The distribution of mucinous and poorly differentiated adenocarcinomas is significantly higher in patients with full methylation compared to those with no methylation. In genotype G/G, there was no difference among the three methylation types except for MSI-H.

### *MLH1*genotypes and other risk factors contributing to *MLH1*promoter methylation in CRCs

In logistic regression analysis using the genotype of the *MLH1*-93G/A SNP (A/A vs. A/G + G/G), tumor location (right vs. left), gender (female vs. male) and age at onset (more than 70 vs. less than 70) as the independent variables, genotype was shown to be the most significant risk factor for hypermethylation of the *MLH1* promoter region (Table 3). The odds ratio (OR) in a multivariate analysis for genotype A/A was 2.82 (95% CI 1.42–5.62, p = 0.003), followed by tumor location (OR 2.4, 95% CI 1.23–4.70, p = 0.011) and gender (OR 2.13, 95% CI 1.08–3.92, p = 0.027), while age at onset was not a significant factor contributing to methylation status.

**Table 3:** **Multivariate**-**adjusted odds ratio for** ***MLH1*** **promoter methylation with genotypes and other variables**

Factors	OR ^1^	95% CI ^2^	p values ^3^
Genotype of the *MLH1* (A/A vs. A/G, G/G)	2.82	1.42-5.62	0.003
Tumor location (right vs. left)	2.40	1.23-4.70	0.011
Gender (female vs. male)	2.06	1.08-3.92	0.027
Age of onset (more than 70 vs. less than 70)	1.05	0.53-2.07	0.885

^1^ *OR*: Odds ratio, Odds ratio adjusted for other variables listed in the table.

^2^ *CI*: Confidence interval.

^3^P values of <0.05 were statistically significant.

### Analysis of genotype frequencies and haplotype of the SNPs surrounding the *MLH1*-93G/A SNP and their association with methylation status

We analyzed the genotype frequencies of six SNPs located -47 kb upstream to 6.4 kb downstream of the MLH1 translation start site, namely, rs2276807, rs4678922, rs6789043, rs1046512, rs3774343 and rs4647215, surrounding the *MLH1*-93G/A SNP (rs1800734) (Figure 2a), and their association with *MLH1* methylation status (methylation-positive vs. methylation-negative). The observed frequency of each genotype was consistent with the expected frequency according to the Hardy-Weinberg equilibrium. There were no significant associations between the frequency of each genotype and the methylation status in these SNPs, except for rs1800734. Furthermore, we analyzed the haplotypes of these seven SNPs and their association with *MLH1* methylation status (methylation-positive vs. methylation-negative). As a result, only the haplotype A-A-T-A-A-A-C (rs2276807-rs4678922-rs6789043-rs1046512-rs1800734-rs3774343-rs4647215) had a significant association with *MLH1* promoter hypermethylation in the analysis of all cases (p = 0.012), as well as in the female cases (p = 0.00038), but this was not significant in the male cases (Table 4).

**Table 4:** **Association between haplotype of seven SNPs including** ***MLH1***-**93G**/**A SNP and the methylation status of the** ***MLH1*** **promoter region**

<Total cases > Haplotype ^1^	Haplotype frequencies ^2^			χ ^2^-value	p-value ^3^
	Total	M-negative	M-positive		
C-A-T-A-**A**-A-C	0.3141	0.3031	0.3431	0.6274	0.4283
A-A-T-A-**G**-A-C	0.3116	0.3366	0.2499	2.9665	0.085
A-A-T-A-**A**-A-C	**0.198**	**0.1667**	**0.2755**	**6.3116**	**0.012**
C-T-C-C-**G**-G-A	0.149	0.1644	0.1102	1.9578	0.1618
C-A-T-A-**G**-A-C	0.0273	0.0291	0.0213	0.1968	0.6573
<Female cases > Haplotype^1)^	Haplotype frequencies^2^			χ^2^-value	p-value^3^
	Total	M-negative	M-positive		
C-A-T-A-**A**-A-C	0.2983	0.3046	0.2955	0.0154	0.9014
A-A-T-A-**G**-A-C	0.2801	0.3346	0.2017	3.3986	0.0653
A-A-T-A-**A**-A-C	**0.2077**	**0.1154**	**0.3452**	**12.6416**	**0.00038**
C-T-C-C-**G**-G-A	0.1829	0.21	0.1406	1.2578	0.2621
C-A-T-A-**G**-A-C	0.0309	0.0354	0.017	0.4818	0.4876
<Male cases > Haplotype^1^	Haplotype frequencies^2^			χ^2^-value	p-value^3^
	Total	M-negative	M-positive		
C-A-T-A-**A**-A-C	0.3323	0.3381	0.3105	0.1457	0.7027
A-A-T-A-**G**-A-C	0.3243	0.3028	0.4031	1.9442	0.1632
A-A-T-A-**A**-A-C	0.1916	0.1922	0.1895	2.00E-03	0.9643
C-T-C-C-**G**-G-A	0.127	0.1414	0.0741	1.7339	0.1879
C-A-T-A-**G**-A-C	0.0249	0.0255	0.0229	0.0117	0.9138

^1^Loci of SNPs examined were as follows: rs2276807 - rs4678922 - rs6789043 -rs1046512- rs1800734 (bold letter) - rs3774343 - rs4647215.

^2^Total cases, methylation-negative (M-negative) cases and methylation-positive (M-positive) cases of haplotype frequencies.

^3^P values of <0.05 were statistically significant.

### EMSA

To elucidate whether the genotype of the *MLH1*-93G/A SNP affects the binding activity of nuclear transcription factors, each synthetic double-stranded DNA oligomer of the *MLH1*-93G/A SNP was subjected to EMSA (Figure 2b). First, in order to confirm the accuracy of this assay, a competition assay was performed using ^32^P-labeled MLH-184 to -132 and unlabeled MLH1-184 to -132 oligomers as described previously [12] (Figure 2b, lanes 1–3). A shifted band was observed with HeLa nuclear extract, which was blocked completely in the presence of an excess of unlabeled competitor sequence, as reported previously. HeLa cell nuclear extract was then incubated with 20-bp DNA oligomers homologous to each genotype of the *MLH1*-93G/A SNP (Figure 2b, lanes 4–7, 8–11). When the ^32^P-labeled MLH1-93G probe was mixed with the nuclear extract, we detected a shifted band, which was blocked by the unlabeled MLH1-93G competitor (Figure 2b, lanes 5 and 6), but not by the unlabeled MLH1-93A competitor (Figure 2b, lane 7). However, a shifted band was not detected when ^32^P-labeled MLH1-93A probe was mixed with the HeLa cell nuclear extract (Figure 2b, lanes 9–11).

## Discussion

Methylation of the *MLH1* promoter region and subsequent transcriptional silencing have been shown to play a critical role in the development of MSI-positive CRCs [3]. In a previous study, we reported two types of methylation profile in the *MLH1* promoter region, namely, full methylation and partial methylation [6]. Only the former was associated with *MLH1* gene silencing followed by the MSI-H phenotype. Partial methylation was observed in CRCs that developed in both proximal and distal colon, while the majority of cases showing full methylation developed in the former. The average age at onset was 10 years earlier in CRCs with partial methylation than in cases with full methylation [7]. Partial methylation of the *MLH1* promoter region implies initiation of the methylation process, but only a subset of cases with partial methylation may eventually progress to full methylation and develop MSI-positive CRCs. It is therefore important to elucidate factors contributing to methylation of the *MLH1* promoter region in CRC tumorigenesis.

Recent studies have reported such a factor. The *MLH1*-93G/A SNP located in the promoter region is significantly associated with *MLH1* promoter methylation followed by loss of MLH1 protein expression and plays an important role in MSI-H colorectal tumorigenesis [14, 16–21]. However, these results are derived mostly from Caucasian populations, in which the frequencies of the A-allele and A/A homozygosity are low, at 20–22% and 4–5%, compared with those in East Asians including the Japanese [12, 16, 20, 25]. The allelic frequency of *MLH1*-93A in a Japanese cohort is as much as 50%; therefore, study on the Japanese would be important to validate the results previously reported for Caucasians. The present study confirms the association between the genotype of *MLH1*-93G/A and methylation status for sporadic CRCs in a Japanese population. The frequency of methylation of the *MLH1* promoter region in CRCs is significantly higher in those carrying the A-allele in Japanese. The *MLH1*-93G/A SNP genotype is associated with the methylation status of the *MLH1* promoter region, with either full or partial methylation.

Previous studies reported a different affinity of the DNA binding proteins to the *MLH1*-93G/A SNP, suggesting that some transcription factors bind preferentially to the sequence of MLH1-93G but not to MLH1-93A [26, 27]. In EMSA using HeLa cell nuclear extract, the band showing a mobility shift of the ^32^P-labeled MLH1-93G probe had greater intensity than that of the MLH1-93A probe, suggesting that the affinity of DNA binding proteins to the MLH1-93A sequence is less than that to MLH1-93G (Figure 2b). These results suggest that individuals carrying the *MLH1*-93A allele are prone to transcriptional silencing due to the decreased affinity to allele-specific DNA binding proteins. However, the definitive mechanism of developing *MLH1* promoter methylation based on the different affinity of DNA binding proteins to the *MLH1*-93G/A SNP has not been elucidated yet. Furthermore, the frequencies of MSI-positive CRC were reported to be as high as 10–15% in Caucasians, compared with 9.05% (19/210) in this series and 13% in another Japanese cohort [28]. Assuming that the frequency of MSI in the Japanese is the same as that in Caucasians, carrying the A-allele at the *MLH1*-93G/A SNP is not the only risk factor for developing MSI-positive CRCs. Methylation of the *MLH1* promoter region was found in 28.6% (60/210) of patients and 78.3% (47/60) showed partial methylation, among which methylation was limited to the most upstream region of the *MLH1* promoter sequences that is distant from the *MLH1*-93G/A SNP. As shown in Table 2, the frequency of A/A homozygosity was significantly higher in cases with partial methylation (42.5%, 20/47) than in those with full methylation (30.8%, 4/13) or no methylation (20.0%, 30/150) (p = 0.0229). These data suggest that the *MLH1*-93G/A genotype was not the only determinant of the MSI phenotype, which was mostly associated with full methylation.

We further examined the association between the genotypes of the six other SNPs surrounding the *MLH1*-93G/A SNP (rs1800734) and methylation status in the *MLH1* promoter region. Rs1800734 was the only SNP showing a significant association with methylation. A haplotype-based case–control study showed a significant association of the haplotype comprising A-A-T-A-A-A-C alleles with *MLH1* promoter methylation (Table 4). The A allele of rs1800734 had only 2 haplotypes comprising either C-A-T-A-A-A-C or A-A-T-A-A-A-C, and the latter showed a significant association with *MLH1* promoter hypermethylation; that is, rs2276807 located in the most upstream region spanning -47 kb of rs1800734 was a determinant of the haplotype associated with *MLH1* promoter hypermethylation. These findings suggest that additional genetic and/or environmental factors may contribute to the progression of methylation status from partial to full methylation, eventually associated with the MSI phenotype. To our knowledge, this is the first report of an association between this haplotype and methylation status in the *MLH1* promoter region.

In an analysis of cases subdivided by gender, the frequency of the A-allele *MLH1*-93G/A SNP was significantly higher in females (RR 1.525, p = 0.0067), while this was not significant in males (RR 1.179) (Table 2). Subjects carrying the A-allele, particularly females, may harbor an increased risk of methylation of the *MLH1* promoter region. Furthermore, a haplotype associated with A-alleles of both rs2276807 and *MLH1*-93G/A (rs1800734) also had a significant association with *MLH1* promoter methylation in female subjects (Table 4). Slattery et al. suggested that estrogen exposure in women protects against MSI, whereas the lack of estrogen in older women increases the risk of MSI, as shown in a population-based case–control study [29]. In this series, the clinical characteristics of methylation-positive tumors, such as female predominance and late age at onset, are compatible with this epidemiological observation for MSI-positive tumors. Individuals carrying the A-allele of *MLH1*-93G/A (rs1800734), especially in association with the A-allele of rs2276807, may be susceptible to methylation, but hormonal status or other genetic factors may modify the age of onset of the cancer. As the present study size is relatively small, further studies are needed to elucidate the mechanism of methylation susceptibility of the *MLH1* promoter region defined by the genotype of *MLH1*-93G/A SNP, along with the relevant haplotype.

## Conclusions

The *MLH1*-93G/A SNP (rs1800734) was significantly associated with methylation of the *MLH1* promoter region in a Japanese population. Furthermore, a haplotype comprising the A-allele of rs2276807 and the A-allele of *MLH1*-93G/A SNP showed a significant association with methylation of the *MLH1* promoter region. Individuals, particularly females, carrying the A-allele at the *MLH1*-93G/A SNP, especially in association with the A-allele of rs2276807, may harbor an increased risk of methylation of the *MLH1* promoter region.

## Funding

This work was supported in part by Grants-in-Aid for Cancer Research and for the Third Term Comprehensive Control Research for Cancer from the Ministry of Health, Labor and Welfare, Japan, and a Grant-in-Aid for Young Scientists B (No. 17790925) from the Ministry of Education, Culture, Sports, Science and Technology, Japan, as well as a Jichi Medical School Young Investigator Award.

## Electronic supplementary material

Additional file 1: **Haplotype defined by the MLH1-93G/A polymorphism. Table S1.** PCR primer sequences and PCR conditions of the six SNPs. **Table S2.** Association between clinicopathological features and methylation status according to the genotype of the *MLH1* promoter region. (DOC 64 KB)

## Abbreviations

BiPS: Na-bisulfite PCR/single-strand conformation polymorphism
CRC: Colorectal cancer
EMSA: Electrophoretic mobility shift assay
HNPCC: Hereditary nonpolyposis colorectal cancer
*MMR* gene: mismatch repair gene
MSI: Microsatellite instability
MSI-H: high-frequency MSI
MSI-L: low-frequency MSI
MSP: Methylation-specific PCR
PBL: Peripheral blood lymphocyte
SNP: Single-nucleotide polymorphism
SSCP: Single-strand conformation polymorphism.
