# Supplementary material for: Haplotype defined by the MLH1-93G/A polymorphism is associated with MLH1 promoter hypermethylation in sporadic colorectal cancers
Source: BMC Res Notes. 2014 Nov 24;7:835. doi: 10.1186/1756-0500-7-835 (PMC4253604; doi:10.1186/1756-0500-7-835)
Supplement: Supplementary file 1 — Additional file 1: Haplotype defined by the MLH1-93G/A polymorphism. Table S1. PCR primer sequences and PCR conditions of the six SNPs. Table S2. Association between clinicopathological features and methylation status according to the genotype of the MLH1 promoter region. (DOC 64 KB) [file 13104_2013_3352_MOESM1_ESM.doc]

Supplementary Table 1. PCR primer sequences and PCR conditions of the six SNPs

| SNP | | Forward(F)/Reverse(R) | | Primer sequence | | | | |  |
| --- | --- | --- | --- | --- | --- | --- | --- | --- | --- |
| rs2276807 | | F | | GGGAGTGAAGGGGGCAGCGAGAC | | | | |  |
|  | | R | | CGATCATCACTGGGGAGGTGGAAAGA | | | | |  |
| rs4678922 | | F | | AACAATAGTAATTCTTCCAGTCCA | | | | |  |
|  | | R | | AAGTGAAAGATCTATAGAAGGACA | | | | |  |
| rs6789043 | | F | | TCTCTTTTAAGGACAAGGCCACAT | | | | |  |
|  | | R | | ATACCCTTTATCCCTTTCCCTGTT | | | | |  |
| rs1046512 | | F | | TTAGACAAAACACCTCCAC | | | | |  |
|  | | R | | GTCTTAAATTATCAACTCCTT | | | | |  |
| rs3774343 | | F | | CGCACTTCGAAGATACCAGATTTT | | | | |  |
|  | | R | | TGGGCTTGTCTTTTTGGAAGTTTA | | | | |  |
| rs4647215 | | F | | CACTCTGTCGCCCACACTGGAG | | | | |  |
|  | | R | | AGGCATGGTGGTGTGAACCTACAG | | | | |  |
| SNP | PCR product size  (bp) | | Gel concentration  (%) | | Electrophoresis time (minute) | Temperature  (°C) | Voltage  (V) | Instruments | |
| rs2276807 | 114 | | 15 | | 900 | 16 | 20 watts^1)^ | ALF^1)^ | |
| rs4678922 | 108 | | 15 | | 300 | 9 | 250 | MiniGel | |
| rs6789043 | 108 | | 15 | | 210 | 9 | 250 | MiniGel | |
| rs1046512 | 118 | | 15 | | 210 | 9 | 250 | MiniGel | |
| rs3774343 | 138 | | 15 | | 450 | 6 | 200 | MiniGel | |
| rs4647215 | 128 | | 8 | | 60 | 22 | 200 | MiniGel | |

1. Electrophoresis was performed at 20 watts by using ALF (ALFexpress DNA sequencer (Pharmacia, Tokyo, Japan))

Supplementary Table 2. Association between clinicopathological features and methylation status according to the genotype of the *MLH1* promoter region

|  | *MLH1*-93 G/A, Genotype A/A (n=54) | | | | | | *MLH1*-93 G/A, Genotype A/G (n=108) | | | | | | *MLH1*-93 G/A, Genotype G/G (n=48) | | | | | |
| --- | --- | --- | --- | --- | --- | --- | --- | --- | --- | --- | --- | --- | --- | --- | --- | --- | --- | --- |
|  | Full  methyl^4)^  n=4 | Partial methyl  n=20 | No  methyl  n=30 | p^5)^  F vs N | p^6)^  F vs P | p^7)^  P vs N | Full  methyl  n=8 | Partial methyl  n=18 | No  methyl  n=82 | p^5)^  Fvs N | p^6)^  F vs P | p^7)^  P vs N | Full methyl  n=1 | Partial methyl  n=9 | No  methly  n=38 | p^5)^  Fvs N | p^6)^  F vs P | p^7)^  P vs N |
| Age at Onset  Total  Male  Female  Gender  Male  Female  Location^1)^  Right  Left  Pathology^2)^  well/mode  muc/por  Dukes  A/B  C/D  Tumor size^3)^  MSI  MSI-H  MSI-negative | 82.0±5.5  -  82.0±5.5  0  4  4  0  1/2  0/1  0/4  0/0  68.3±51.6  4  0 | 67.5±8.3  68.2±8.3  66.7±8.7  10  10  6  14  13/6  0/1  1/13  6/0  63.0±29.0  1  19 | 59.4±7.8  60.0±7.8  57.9±8.3  21  9  6  24  23/4  1/2  2/14  12/2  51.2±19.6  1  29 | <0.0001  -  0.0003  0.007  0.001  NS  NS  NS  <0.0001 | 0.003  -  0.007  NS  0.01  NS  NS  NS  <0.0001 | 0.001  0.011  0.038  NS  NS  NS  NS  NS  NS | 70.5±8.9  68.7±5.0  71.6±11.0  3  5  8  0  4/1  2/1  1/5  1/1  68.1±30.8  8  0 | 63.7±10.1  61.3±10.36  66.6±9.6  10  8  6  12  8/8  2/0  1/10  6/1  52.2±27.9  1  17 | 65.0±11.5  64.0±11.7  67.5±10.8  58  24  24  58  58/19  4/1  4/37  29/12  52.9±23.1  1  81 | NS  NS  NS  NS  <0.0001  0.02  NS  NS  <0.0001 | NS  NS  NS  NS  0.0016  NS  NS  NS  <0.0001 | NS  NS  NS  NS  NS  NS  NS  NS  NS | 55.0  -  55.0  0  1  1  0  1/0  0/0  0/1  0/0  45.0  1  0 | 59.6±12.7  64.2±14.0  53.8±9.3  5  4  3  6  3/5  1/0  0/4  2/3  59.4±19.4  0  9 | 65.2±11.7  65.8±10.9  64.5±13.0  21  17  9  28  24/13  0/1  2/24  10/2  51.7±23.2  2  36 | -  -  -  NS  NS  NS  NS  -  0.0004 | -  -  -  NS  NS  NS  NS  -  0.002 | NS  NS  NS  NS  NS  NS  NS  NS  NS |

1) right: cecum, ascending colon and transverse colon, left: descending colon, sigmoid colon and rectum.

2) well: well-differentiated adenocarcinoma, mod: moderately differentiated adenocarcinoma, por: poorly differentiated adenocarcinoma, muc: mucinous adenocarcinoma.

3) Unit are in mm

4) methyl: methylation

5) p values were analyzed using Pearson's chi-square test or Student's t-test between patients with F (full methylation) and patients with N (no methylation). NS: not significant. A p-value <0.05 is statistically significant.

6) p values were analyzed using Pearson's chi-square test or Student's t-test between patients with F (full methylation) and patients with P (partial methylation). NS: not significant. A p-value <0.05 is statistically significant.

7) p values were analyzed using Pearson's chi-square test or Student's t-test between patients with P (partial methylation) and patients with N (no methylation). NS: not significant. A p-value <0.05 is statistically significant.
